# Incidence and Antenatal Detection of Congenital Heart Malformations—Data from a Tertiary Obstetric Romanian Center

**DOI:** 10.3390/diagnostics14151659

**Published:** 2024-08-01

**Authors:** Adrian Ciulpan, Adrian Lacatușu, Liviu Laurenţiu Pop, Corina Paul, Diana Lungeanu, Daniela Iacob, Brenda-Cristiana Bernad, Ana Lascu, Edida Maghet, Diana-Aurora Arnautu, Elena Silvia Bernad

**Affiliations:** 1 Doctoral School, “Victor Babeș” University of Medicine and Pharmacy, 300041 Timișoara, Romania; ciulpan.adrian@umft.ro (A.C.); bernad.brenda@umft.ro (B.-C.B.); 2 IInd Pediatrics Clinic, “Pius Brinzeu” County Clinical Emergency Hospital, 300723 Timișoara, Romania; pop.liviu@umft.ro (L.L.P.); paul.corina@umft.ro (C.P.); 3 Department of Pediatrics, “Victor Babeș” University of Medicine and Pharmacy, 300041 Timișoara, Romania; 4 Center for Modeling Biological Systems and Data Analysis, “Victor Babeș” University of Medicine and Pharmacy, 300041 Timișoara, Romania; dlungeanu@umft.ro; 5 Department of Functional Sciences, Faculty of Medicine, “Victor Babeș” University of Medicine and Pharmacy, 300041 Timișoara, Romania; lascu.ana@umft.ro; 6 Department of Obstetrics and Gynecology, “Victor Babeș” University of Medicine and Pharmacy, 300041 Timișoara, Romania; iacob.daniela@umft.ro (D.I.); bernad.elena@umft.ro (E.S.B.); 7 Clinic of Neonatology, “Pius Brinzeu” County Clinical Emergency Hospital, 300723 Timișoara, Romania; 8 Center for Neuropsychology and Behavioral Medicine, “Victor Babeș” University of Medicine and Pharmacy, 300041 Timișoara, Romania; 9 Multidisciplinary Heart Research Center, “Victor Babeș” University of Medicine and Pharmacy, 300041 Timișoara, Romania; aurora.bordejevic@umft.ro; 10 Institute of Cardiovascular Diseases Timișoara, 300310 Timișoara, Romania; 11 Center for Translational Research and Systems Medicine, “Victor Babeș” University of Medicine and Pharmacy, 300041 Timișoara, Romania; 12 Ist Department, Faculty of Dental Medicine, “Victor Babeș” University of Medicine and Pharmacy, 300041 Timișoara, Romania; edidamaghet@gmail.com; 13 Department of Internal Medicine, “Victor Babeș” University of Medicine and Pharmacy, 300041 Timișoara, Romania; 14 Ist Clinic of Obstetrics and Gynecology, “Pius Brinzeu” County Clinical Emergency Hospital, 300723 Timișoara, Romania; 15 Center for Laparoscopy, Laparoscopic Surgery and In Vitro Fertilization, “Victor Babeș” University of Medicine and Pharmacy, 300041 Timișoara, Romania

**Keywords:** heart, congenital malformation, incidence, prenatal ultrasound, diagnostic

## Abstract

Objectives: Congenital heart defects (CHDs) are among the most frequent congenital defects, and they significantly burden the healthcare system due to their high mortality rate and high cost of care for survivors. We aimed to highlight the incidence of CHDs in a tertiary center in Western Romania. Methods: A retrospective study was carried out between 2018 and 2022 at the “Pius Brinzeu” Emergency County Hospital Timisoara. Relevant information about the mothers and the newborns were collected and statistically analyzed. Results: The incidence of CHDs from 2018 to 2022 in our center was 5.3%. Eleven types of malformations were diagnosed postnatally in 541 newborns, with 28.8% of cases having more than one type of CHD. The antenatal detection rate was 28%, with the highest rates for tetralogy of Fallot, hypoplastic left heart syndrome, or significant ventricular septal defects and the lowest for pulmonary stenosis. The lower antenatal detection rate was influenced mainly by incomplete or absent prenatal care. Conclusions: The incidence of CHDs is clearly dependent of a multifactorial approach, and the results highlight this. With an incidence almost 50% lower than reported within the literature and a low rate of prenatal detections, CHDs could be a more of a burden to endure regarding medical treatment. Improvements in patients’ education, prenatal care, and screening programs could improve diagnosis, decrease mortality, and optimize postnatal care.

## 1. Introduction

The role of epidemiology in public health is undiscussable nowadays, mainly when we refer to diagnostics with a high degree of morbidity and mortality, like congenital heart disease (CHD). Despite techno-medical advances, the antenatal and postnatal diagnostics of fetuses and newborns with cardiac malformations are still a challenge, especially in middle- and low-resource countries [1,2,3].

The generally accepted epidemiological description of CHDs as follows. Prevalence: The prevalence of CHDs has been reported to be around 8.2 per 1000 live births globally [1,2,3], increasing up to 10 per 1000 live births [4]. Mortality: Mortality rates associated with CHDs have decreased over the recent few decades due to advancements in surgical techniques, medical management, and early diagnosis [5]. Prenatal Diagnosis: There has been an increase in the prenatal diagnosis of CHDs due to improvements in ultrasound technology and prenatal screening programs [6]. Survival Rates: With advances in pediatric cardiology and cardiac surgery, the survival rates for infants born with CHDs have improved significantly [7]. Risk Factors: Certain risk factors for CHDs, such as maternal age, genetic factors, environmental exposures, and maternal health conditions (such as diabetes or obesity), continue to be areas of research and concern [8].

Unfortunately, the relationship between etiology and specific lesions has not been well studied. A variety of cumulative factors, like infections, drugs, alcohol, tobacco, genetic syndromes, and maybe the quality of air, food, and water are considered risk factors for cardiac malformations [9]. However, in the majority of patients, CHDs appear spontaneously without unidentifiable risk factors, so primary prevention is not possible; thus, their prevalence cannot be modified [10]. The burden of changing this situation lies on secondary prevention methods like antenatal diagnostics. Antenatal diagnosis of CHDs is based on fetal echocardiography, which facilitates two ways of secondary implementation. First, it could set up parental counseling regarding the CHD diagnosis, prognosis, management, and treatment, followed by the pregnant woman’s decision to continue or terminate the pregnancy. Second, it facilitates obstetrical and neonatal approaches for better management and outcomes, improving morbidity and mortality in those cases. Antenatal diagnostic detection varies widely across countries, mainly due to screening policies, available resources, and access to prenatal care [11]. Reported rates range from 25% to as high as 75%. Rates also vary widely between anomalies, with higher rates for those affecting the four-chamber view and lower rates for outflow tract anomalies [12]. Nowadays, it is a challenge, but we hope that in the future the range of risk factors for the development of CHDs that we will be able to identify will be larger and sufficient for lowering epidemiological data—a theme of study for now and for the future [13]. The sustained effort of teratology to find and point out the precise moment and risk factor that leads to the development of CHDs is more acute and needed than ever. The burden is mainly represented by multifactorial complexity; in some cases, it is a genetic susceptibility, but it seems that isolated lesions occur in more than half of the situations—63.6% [14].

In the context of widespread variability in the efficacy of screening programs, we aimed to evaluate the current epidemiology of congenital heart defects in our center along with antenatal detection rates.

## 2. Materials and Methods

This retrospective study was conducted at a tertiary hospital, “Pius Brînzeu” Emergency County Hospital (PBCCEH), Timișoara, Romania, in the Clinic Institute of Obstetrics, Gynecology and Neonatology, over a period of four years (2018–2022). The population included 10,113 births registered in our clinic, which provides obstetrical care for several counties in Western Romania.

The necessary data were collected from the hospital’s electronic database. They included sociodemographic characteristics of the mothers, obstetrical history, prenatal diagnosis data, the evolution of the pregnancies, birth outcomes, the presence of congenital cardiac malformations diagnosed in the period of pregnancy or after birth, and types of malformations. Fetal echocardiography involves obtaining specific views or images of the fetal heart from different angles to thoroughly evaluate its structure and function [15]. These views allow healthcare providers to assess various aspects of the fetal heart and detect any abnormalities. The importance of sonographic views and the amount of anatomic information, as well as relations between various cardiac structures and adjacent ones, are dependent on a multitude of factors (the experiences of the sonographer, the mother’s comfort or discomfort, the fetal positions in utero, the performances of the echo machine, etc.). However, it seems that the consequent use of systematized views is significantly reducing the rates of errors. The most used ones are as follows. Four-Chamber View: This view provides a panoramic image of all four chambers of the fetal heart. Outflow Tract Views: These views focus on the blood vessels that carry blood out of the heart. Three-Vessel and Trachea View (3VT): This view provides an overview of the aorta, pulmonary artery, and superior vena cava, along with the trachea. Short Axis Views: These views provide detailed images of the ventricles, including the mitral and tricuspid valves, and allow for the assessment of ventricular size, wall motion, and valve function. Long Axis Views: These views provide a profile of the heart and allow for visualization of structures such as the aorta, pulmonary artery, and atrioventricular valves. Ductal and Aortic Arch Views: These views focus on the ductal arch and aortic arch to assess their size, shape, and branching pattern. Venous Connections: views of the venous connections, including the superior and inferior vena cava, are obtained to assess their location, size, and connections to the heart. Depending on the clinical indication and suspected abnormalities, additional views may be obtained to comprehensively evaluate the fetal heart.

Our study included, as a prenatal diagnosis, any congenital cardiac defect observed prenatally at an ultrasound scan, routine, or screening.

The PBCCEH Research Committee and the University of Medicine and Pharmacy “Victor Babes” Timisoara Human Research Ethics Committee both approved this study.

Statistical analysis was performed using Jamovi software v2.3.28 [16], and regression analysis, principal component analysis, and factor analysis were used to verify the model’s reliability and validity. Descriptive statistics were applied to all relevant data.

## 3. Results

From the 10,113 births registered during the four years analyzed, 541 newborns from 541 mothers were diagnosed postnatally with a type of congenital cardiac malformation, resulting in an incidence of 5.35%.

### 3.1. Mothers’ Characteristics in Our Study: Physiological Characteristics

The mothers’ ages ranged between 17 and 45, with a mean of 29.4 years. Parity was between 0 and 5 births.

Regarding medical history, unfortunately, the rate of mothers without any prenatal care or with <3 prenatal visits was high, about 38%. In total, 62% of the mothers came from rural areas and were the predominant patients without complete prenatal care.

### 3.2. Mothers’ Characteristics in Our Study: Pathological Characteristics

Among those with a known history or complete prenatal care, 1.9% of the mothers were diagnosed with a pre-existing pathology, most commonly diabetes mellitus type 2 and chronic hypertension. In comparison, 5.4% had a pregnancy-associated pathology, most commonly gestational diabetes and gestational hypertension. Moreover, 0.9% of the included pregnancies were obtained by artificial reproduction techniques (Table 1).

### 3.3. Newborns’ Characteristics

#### 3.3.1. Newborns’ Characteristics in Our Study: Physiological Characteristics

Among the 541 newborns, 52% were female and 48% were male. Gestational age at birth ranged from 25 weeks to 41 weeks, with a mean of 36.2 weeks. The birth weight ranged from 600 g to 5050 g, with a mean of 2747 g. The mean APGAR score at birth was 7. The premature newborn rate in our population was 36%. The leading causes of preterm births were preterm labor, followed by iatrogenic births in cases of intrauterine growth restriction, mostly due to poorly controlled maternal systemic hypertension.

#### 3.3.2. Newborns’ Characteristics in Our Study: Pathological Characteristics

**Figure 1:**
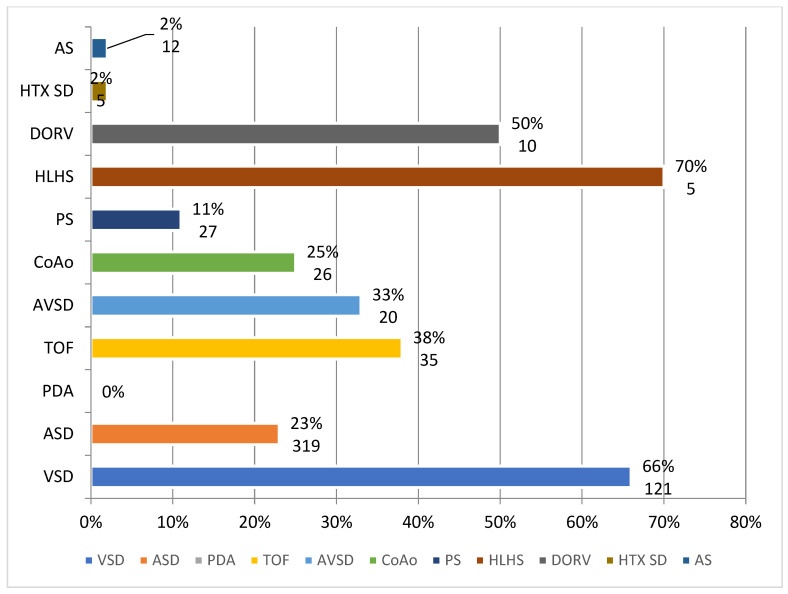
Postnatal diagnosed malformations and the rates of detection in our center. Abbreviations: VSD = ventricular septal defect; ASD = atrial septal defect; PDA = patent ductus arteriosus; TOF = tetralogy of Fallot; AVSD = complete atrioventricular canal defect; CoAo = coarctation of the aorta; PS = pulmonary stenosis; HLHS = hypoplastic left heart syndrome; DORV = double-outlet right ventricle; HTX SD = heterotaxy syndrome; AS = aortic stenosis. Data are presented in percentages for detection rates and numbers for patients.

Eleven types of cardiac malformations were diagnosed postnatally, accounting for 541 individual malformations (Figure 1). Of all 541 patients, only 151 had a prenatal diagnosis of CHD.

The three most common malformations were atrial septal defects, ventricular septal defects, and persistent ductus arteriosus. In patients born with ASDs, we needed to specificize and differentiate the abnormal communications between atrial chambers (counted in our study lot) from Patent Foramen Ovale, who was not counted but was present in a very high number. In the settings of PDA, because of the nature of this study, we could not re-evaluate this condition, so it was important to consider that these numbers were mixed (maybe over-rated).

In 28.8% of newborns (*n* = 156), more than one type of malformation was diagnosed, with the main malformation most frequently being associated with atrial or ventricular septal defects. The prevalence of each malformation type is presented in Table 2. The rate of prenatal diagnosis was 28%, mainly due to the high rate of pregnancies without any prenatal care. Among those with a prenatal diagnosis, most were diagnosed with complex malformations like tetralogy of Fallot, hypoplastic left heart syndrome, or significant ventricular septal defects. In 5% of cases, an associated anomaly was also diagnosed, most frequently involving the central nervous system. Also, 0.8% had a chromosomal anomaly, all with Down syndrome.

**Figure 2:**
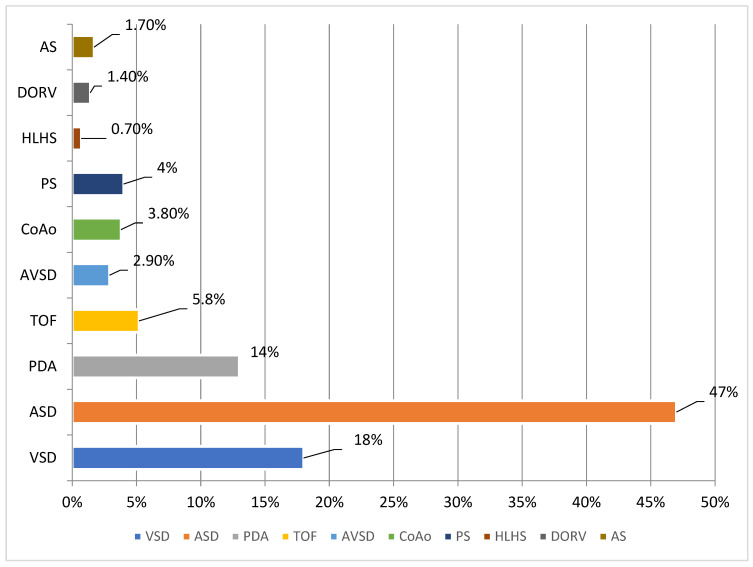
Incidence of congenital cardiac malformations in our center. Abbreviations: VSD = ventricular septal defect; ASD = atrial septal defect; PDA = patent ductus arteriosus; TOF = tetralogy of Fallot; AVSD = complete atrioventricular canal defect; CoAo = coarctation of the aorta; PS = pulmonary stenosis; HLHS = hypoplastic left heart syndrome; DORV = double-outlet right ventricle; AS = aortic stenosis. Data are presented in percentage for incidence and numbers for patients.

The incidence of CHD per live birth has increased in recent decades, mainly due to improvements in prenatal diagnosis and advances in pediatric cardiovascular surgery; thus, in our center (Figure 2), it seems to be lower than in the general reported data [1,2].

## 4. Discussion

Our study investigated the incidence of congenital heart defects in children born in our hospital between 2018 and 2022. As a tertiary center, our hospital provides obstetrical and neonatal care for patients from several counties in Western Romania, being one of the two leading hospitals in a large area caring for high-risk pregnancies and admitting preterm labors under 34 weeks of gestation.

CHDs represent a significant burden on public health worldwide. They are a major cause of mortality in low- and middle-income countries and are associated with high morbidity and high overall costs of care in high-resource countries [17,18]. Reported incidences vary widely, with significant differences between regions. The reason for this variability is unknown, with presumed environmental and genetic factors. Thus, the reported incidence differences are attributed to the variable antenatal diagnosis rates [1].

In Europe, the reported incidence by the European Surveillance of Congenital Anomalies (EUROCAT) was 2.39% [19]. Our center identified an incidence of 5.08%. A few published papers have reported CHD incidences in Romania. In a paper from the Târnăveni area, including births from a secondary hospital with the city, the reported incidence was 0.75%. In comparison, another paper from a large hospital in Cluj-Napoca reported a CHD incidence of 5.7% in newborns [20,21]. The difference can be explained by the complexity of the cases admitted to the investigated hospitals. Our incidence is similar to that of the Cluj-Napoca Hospital, with both hospitals being tertiary centers. It is possible that the large differences between our results and general European reported data (almost a 50% difference) can be explained by the fact that there is only one surgical center in Romania (Targu Mures) that offers treatment for CHDs, and the vast majority of suspected diagnostics in utero are oriented to it. The antenatal diagnosis rate is also widely variable, directly influenced by the rates of prenatal care in the pregnant population, the type of malformation, and screening programs [22,23,24]. Reported rates across Europe vary from 8% in countries from Eastern Europe (Croatia, Lithuania) to 48% in France, with rates as high as 79% in individual European centers [25]. Types of insurance, lower socioeconomic factors, and rural patients are associated with lower prenatal diagnostic rates [26]. Our prenatal overall rate is low (Figure 2), at 28%, but in a population with only 62% complete prenatal care. Incomplete or no prenatal care is mostly explained by a low socio-economic status or a lack of individual medical education. Regarding screening programs, countries with no organized screening reported rates of diagnosis of 17.9%, while countries where several ultrasounds (>3) are routinely performed had prenatal diagnosis rates of 55.5% [27]. The impact of low prenatal detection rates in Romania can first be identified in almost every maternity ward, mostly in emergencies, when CHDs appear in the form of ductal dependency and the vital risk is higher. Secondly, it can be seen when long-term management requires 2–3 surgical interventions and specific medical treatment for complications in the form of economic distress for the family and medical care team.

In Romania, pregnant women attending complete prenatal care benefit from a median of five ultrasounds throughout their pregnancy. Also, the sonographer’s skills are an essential determinant of the detection rate. Although professional ultrasound international associations define indications for fetal echocardiography, most CHDs diagnosed by expert sonographers are referrals for suspected CHDs after regular ultrasounds of low-risk populations and not by referring pregnant women with the mentioned fetal and maternal risk factors for CHDs [28]. Thus, the chance of diagnosing many CHDs is reduced. Computer-aided learning applications have wide applications in the medical field [29,30]. Using such applications could increase the number of specialists who can suspect cardiac malformations and refer the cases to centers specializing in fetal cardiac pathology [31].

The level of education, in all of its forms, must be a very important and identifiable factor in downsizing the high rates of CHDs’ incidence worldwide, especially in developing countries. The maternal level of health and social resources can be easily evaluated; consequently, changes could be made for appropriate maternal–fetal evaluations, but only if they are addressed by those mothers. Unfortunately, the lowest rates for accessing obstetrical evaluations are associated with teen mothers, low social resources, multiparity, abuse of substances (alcohol, drugs, antidepressive medications, etc.), and domestic violence. In those cases, medical advice, evaluations, and care are useless; moreover, in most of them, the mother is admitted to hospital emergency units like a birth emergency, making this type of situation only being able to lead to early diagnostics for CHDs, but with no history of prenatal detection and no strategy for cases with duct-dependent CHDs. Sadly, these facts are common across various developing countries, and these limitations are becoming more and more politically dependent as fair health strategies are associated with higher costs.

The type and magnitude of malformations could be another influencing factor in prenatal diagnosis rates. In some cases, interrupting a pregnancy is medically advised, and the final results depend on the mother’s choice (interruption or continuation of pregnancy with medical and surgical support). Communicating the diagnosis of fetal malformations can induce a complementary state of stress or depression in the patient that can manifest itself both in the prenatal and postpartum periods [32]. Therefore, the diagnosis and fetal prognosis must be communicated only by the specialist who identified the malformation, and if necessary, he should call on the services of a psychologist [33]. The ethics and medical advice behind this situation can be very sensitive; therefore, the best choice for continuations or interruptions of a pregnancy lies between two extremities. On the one hand, for every type of cardiac malformation, however bizarre it is (from an anatomical point of view), someone has created a way to correct or treat it (anatomically or sometimes non-anatomically but in a hemodynamically efficient manner). The other side behind the ethics of this problem is represented by the outcome and the price for survival in terms of life quality and expectancies. Sometimes the magnitude of such a diagnosis could increase the rates of divorce and family separations. Nevertheless, for medical specialists, it is very important to communicate the diagnosis and the magnitude of such a situation in a very objective and clear manner, with precise pros and cons.

Historically, the recommended view for cardiac ultrasound was the “four-chamber” view [34]. This strategy, although able to diagnose many CHDs, also missed many CHDs. Thus, current guidelines updated their recommendation by adding the imaging of the outflow tracts to the basic cardiac imaging views [35]. However, despite all the improvements, the detection rates of many CHDs remain low, especially for those affecting the outflow tracts, which remain suboptimal [8]. As reported in the literature, in our center, most prenatal CHD diagnoses were made for defects involving “the four-chambers view.” Recent studies highlight the fact that the number of CHD cases detected in adulthood is high [36,37]. It has also been demonstrated that half of CHD cases remain undiagnosed immediately after birth [38,39]. Therefore, the echocardiography screening of CHDs in fetuses and neonates is crucial to establishing the diagnosis on time and facilitating the patient’s access to the appropriate treatment promptly [40,41]. In cases of congenital cardiac malformations diagnosed in the prenatal period, referring the case to a center specializing in pediatric cardiac surgery is essential. Suppose it is estimated that the malformation type requires surgical intervention immediately after birth. In that case, it is recommended that the birth to be scheduled and for the birth to occur in a medical unit near the surgical center [42].

### Limitations of This Study

One main limitation of the current study is the small sample size as patient data were inoperable regarding the main outlined regulations. Many of these patients were provided by the neonatology unit, and the lack of medical regulations for cardiac diagnostics significantly reduced the sample size for this study.

Another limitation of this study was generated in some cases by the selections made for severe cardiac malformations (duct dependency) in utero by the fetal sonographers who were redirected for surgically annexed maternities, so the data provided cannot uniformly cover all types of CHDs in this statistical representation.

Some specific parameters in parents, like genetics, substance abuse, and medications, were unknown (and were not represented in medical files). Thus, this kind of information was inconclusive for this study in terms of possible etiology.

## 5. Conclusions

Congenital heart defects are a significant cause of neonatal morbidity and mortality, and their incidence is increasing worldwide. However, in recent decades, prenatal ultrasound has significantly improved the rates of prenatal detection, but CHD prenatal diagnosis remains suboptimal in many settings. Secondary prevention measures such as enhanced patient prenatal education, better screening programs, and improved ultra sonographic skills could increase detection rates, reducing neonatal mortality and enabling optimal postnatal care.

Advancements in medical technology, particularly in the field of prenatal screening and imaging, have significantly contributed to the higher detection rates of cardiac anomalies in utero. Here are some key factors:

Improvements in Ultrasound Technology: High-resolution ultrasound machines with advanced imaging capabilities allow healthcare providers to visualize the developing fetal heart more clearly and enable them to accurately detect abnormalities.

Fetal echocardiography: This specialized ultrasound technique focuses specifically on imaging the fetal heart. It enables a detailed examination of the structure and function of the fetal heart, making it easier to identify cardiac anomalies early during pregnancy.

Increased Awareness and Training: Healthcare providers are now more aware of the importance of screening for cardiac anomalies during prenatal care. There has been an emphasis on training obstetricians, sonographers, and other healthcare professionals to recognize signs of cardiac abnormalities during routine prenatal ultrasound examinations.

Expanded Screening Programs: Many healthcare facilities have implemented routine fetal anatomy scans, including detailed cardiac evaluations, as part of standard prenatal care. This proactive approach increases the likelihood of detecting cardiac anomalies early during pregnancy.

Genetic Testing: Advances in genetic testing techniques, such as non-invasive prenatal testing (NIPT) and amniocentesis, can detect chromosomal abnormalities associated with certain cardiac defects. Identifying these genetic markers can prompt further evaluations of the fetal heart.

Multi-Disciplinary Approach: Collaborative efforts between obstetricians, maternal-fetal medicine specialists, pediatric cardiologists, and other healthcare professionals facilitate the comprehensive evaluation and management of fetal cardiac anomalies. This multi-disciplinary approach ensures that appropriate interventions and treatment plans are developed for affected pregnancies.

Overall, the combination of technological advancements, increased awareness, and collaborative healthcare practices has led to higher detection rates of cardiac anomalies in utero, allowing for early intervention and improved outcomes regarding affected pregnancies.

In the context of cardiac malformations, secondary prevention might also involve specific measures tailored to the particular type of malformation present. For example, in the case of congenital heart defects, secondary prevention could include monitoring for complications like arrhythmias, heart failure, or infective endocarditis and implementing appropriate interventions to manage these risks. The goal is to improve the quality of life for individuals with cardiac malformations and reduce the likelihood of adverse outcomes associated with their condition.

## Figures and Tables

**Table 1:** Patients’ characteristics.

Mothers’ Characteristics	
Variables	*n* = 541
Gestational age [weeks] ^(a),(b)^	36.2 ± 5.51
	36 (25–41)
Maternal age [years] ^(a),(b)^	29.4 ± 5.51
	29 (17–45)
Rural area ^(c)^	335 (62)
Para ^(b)^	2 (0–5)
Mother pre-existent pathology ^(c)^	10 (1.9)
Mother pregnancy-associated pathology ^(c)^	29 (5.4)
Human-assisted reproduction techniques ^(c)^	5 (0.9)
Neonates’ Characteristics	
Birth weight [g] ^(b)^	2747 (600–5050)
Female/Male ^(c)^	281 (52)/260 (48)
Apgar Score ^(b)^	7 (4–9)
Twin pregnancy ^(c)^	10 (1.9)
Premature ^(c)^	195 (36)
Associated anomalies ^(c)^	27 (5)
Chromosomal anomaly ^(c)^	4 (0.8)

^(a)^ mean ± std. dev.; ^(b)^ median; ^(c)^ observed frequency (percentage).

**Table 2:** The incidence and rates of detection of congenital cardiac malformations in our center.

Congenital Heart Defect Type	Incidence (*n* = 541)	Rate of Detection
	%	%
Ventricular septal defect	18	66
Atrial septal defect	47	23
Patent ductus arteriosus	14	N/A
Tetralogy of Fallot	5.8	38
Complete atrioventricular canal defect	2.9	33
Coarctation of the aorta	3.8	25
Pulmonary stenosis	4	11
Hypoplastic left heart syndrome	0.7	70
Double-outlet right ventricle	1.4	50
Heterotaxy syndrome	0.7	2
Aortic stenosis	1.7	2

Note: *n* = 541 participants; N/A—not available.

## Data Availability

Raw data were retrieved from the patients’ records and can be made available only upon formal request and approval of the hospital’s management and ethics committee.
